# Separation of Biological Particles in a Modular Platform of Cascaded Deterministic Lateral Displacement Modules

**DOI:** 10.1038/s41598-018-34958-8

**Published:** 2018-12-10

**Authors:** Eloise Pariset, Charlotte Parent, Yves Fouillet, Boizot François, Nicolas Verplanck, Frédéric Revol-Cavalier, Aurélie Thuaire, Vincent Agache

**Affiliations:** University Grenoble Alpes, CEA, LETI, DTBS, F-38000 Grenoble, France

## Abstract

Deterministic lateral displacement (DLD) has been extensively implemented in the last decade for size-based sample preparation, owing to its high separation performances for a wide range of particle dimensions. However, separating particles from 1 *μ*m to 10 *μ*m in one single DLD device is challenging because of the required diversity of pillar dimensions and inherent fabrication issues. This paper presents an alternative approach to achieve the extraction of *E. coli* bacteria from blood samples spiked with prostate cancer cells. Our approach consists in cascading individual DLD devices in a single automated platform, using flexible chambers that successively collect and inject the sample between each DLD stage without any external sample manipulation. Operating DLD separations independently enables to maximize the sorting efficiency at each step, without any disturbance from downstream stages. The proposed two-step automated protocol is applied to the separation of three types of components (bacteria, blood particles and cancer cells), with a depletion yield of 100% for cancer cells and 93% for red blood cells. This cascaded approach is presented for the first time with two DLD modules and is upscalable to improve the dynamic range of currently available DLD devices.

## Introduction

Since its first introduction in 2004 by Huang *et al*.^1^, Deterministic Lateral Displacement (DLD) has been extensively implemented and improved^2–4^ to purify biological components, while preserving their integrity, such as blood cells^5–8^, circulating tumor cells^9–11^, bacteria^12^, parasites^13^, spores^14^ and even nanoparticles^15–17^. In DLD devices, passive and label-free separation is induced by an array of regularly arranged pillars that give specific trajectories to particles of different sizes. Particles larger than a cutoff diameter (called critical diameter *Dc*) are deviated along the pillar array, while smaller particles display a zigzag trajectory along the channel length. This separation enables to collect two particle subpopulations in separated DLD outlets. The value of the critical diameter can be customized at the design stage by adjusting the geometrical parameters of the pillar array, such as the inter-pillar spacing, slant angle, pillar shape and orientation^18–21^.

In order to purify complex biological samples containing a range of particle sizes, a series of DLD-based separations are executed sequentially. Normally, particles are injected into a single DLD device containing successive pillar arrays with specific geometric characteristics so that each array generates two particle populations - a population above and another below the *Dc*^5,9,13,22,23^. Applying this approach to process a heterogeneous sample containing particles with size scales in the micrometer and nanometer ranges - the typical content of a biofluid sample - would require inter-pillar spacings ranging from a few tens of micrometers^9^ down to the submicron scale^15^. However, etching such range of dimensions on a single silicon wafer is limited by a maximum pillar depth to gap ratio of approximately^20^ (with our fabrication technology) unless several complex intermediary lithography and etching steps are incorporated into the fabrication protocol. Instead, we propose a modular approach by assembling several individual DLD building blocks separately (fabricated on separate wafers each with optimized etching conditions). The most efficient DLD modules and flow rates can then be identified separately before cascading the different devices onto a single microfluidic cartridge to allow for sequential flow. This approach enables a higher sample throughput as the flow rate in the entire device is no longer limited by the most resistive DLD section represented by the smallest inter-pillar spacing. Instead, each DLD device within the cartridge is operated independently and the overall flow rate is not limited by downstream DLD steps.

The principal challenge when connecting a DLD module to downstream microfluidic steps is to keep a constant flow velocity across the different DLD outlets in order not to disturb the trajectory of particles. Karabacak *et al*.^11^ reported a CTC-iChip with a first DLD step connected to a second purification device. Only the DLD outlet that contains large deviated cells is connected to the downstream step, which induces higher hydraulic resistance on this outlet compared to the non-deviated outlet that contains waste blood contaminants. A syringe pump is connected to this waste channel in order to apply a controlled output flow rate and compensate for the resistance imbalance. This solution is appropriate when only one of the two particle subpopulations has to be collected. Otherwise, connecting a syringe pump does not allow to collect particles flowing through this outlet, which is problematic when further purification or analysis is required. Another version of the CTC-iChip was proposed by Fachin *et al*.^24^, which uses a resistive serpentine channel at the waste outlet - instead of a syringe pump - to specifically compensate for the pressure loss at the other outlet (containing CTCs and white blood cells, that are further separated by downstream magnetic purification).

We recently proposed an alternative solution to collect particles from all the different DLD outlets, while balancing output hydraulic resistances^25^, based on droplet generation in T-junctions positioned at the DLD outlets. Droplets enable at the same time to apply specific resistances and encapsulate particles flowing out from the DLD device. However, digital microfluidics is not required for all applications and the use of an immiscible phase - necessary for droplet generation - has to be avoided for some downstream steps.

In this paper we present a new modular approach to cascade independent DLD devices and collect purified particles at each step, without any complex external control (no syringe pumps or biphasic microfluidic approach is involved). In order to avoid degradation of the sorting efficiency - that is usually induced when connecting a DLD outlet to another device - purification steps are performed successively and independently. DLD devices are temporarily isolated during each purification step by valves and collection chambers, that are integrated in a common platform and automatically actuated without any requirement for intermediary sample manipulation. The implemented chambers are flexible and collapsible with the help of a hyperelastic material. The advantage of this new type of chamber is to avoid air injection in the microfluidic channels in order to minimize bubble formation that is critical for DLD separation. Given the high elasticity of the membrane, we achieve the storage of sample volumes of up to 1 mL in our cartridge between the different DLD stages. These chambers have a dual function for both collection and injection steps in order to transfer the sample from one DLD stage to the subsequent module.

After validation of the autonomous platform with model particles, our cascaded separation solution was applied to the fractionation of three main particle dimensions (*E. coli* bacteria, Red Blood Cells (RBCs) and prostate cancer cells) in order to isolate bacteria from a complex blood sample, which could open perspectives towards sepsis diagnostics, after improvements of the device throughput. Thus, this paper reports a new approach to process biological samples through several cascaded purification steps, with the potential of strongly improving achievable dynamic ranges of DLD sorting.

## Methods

### Devices

**Figure 1:**
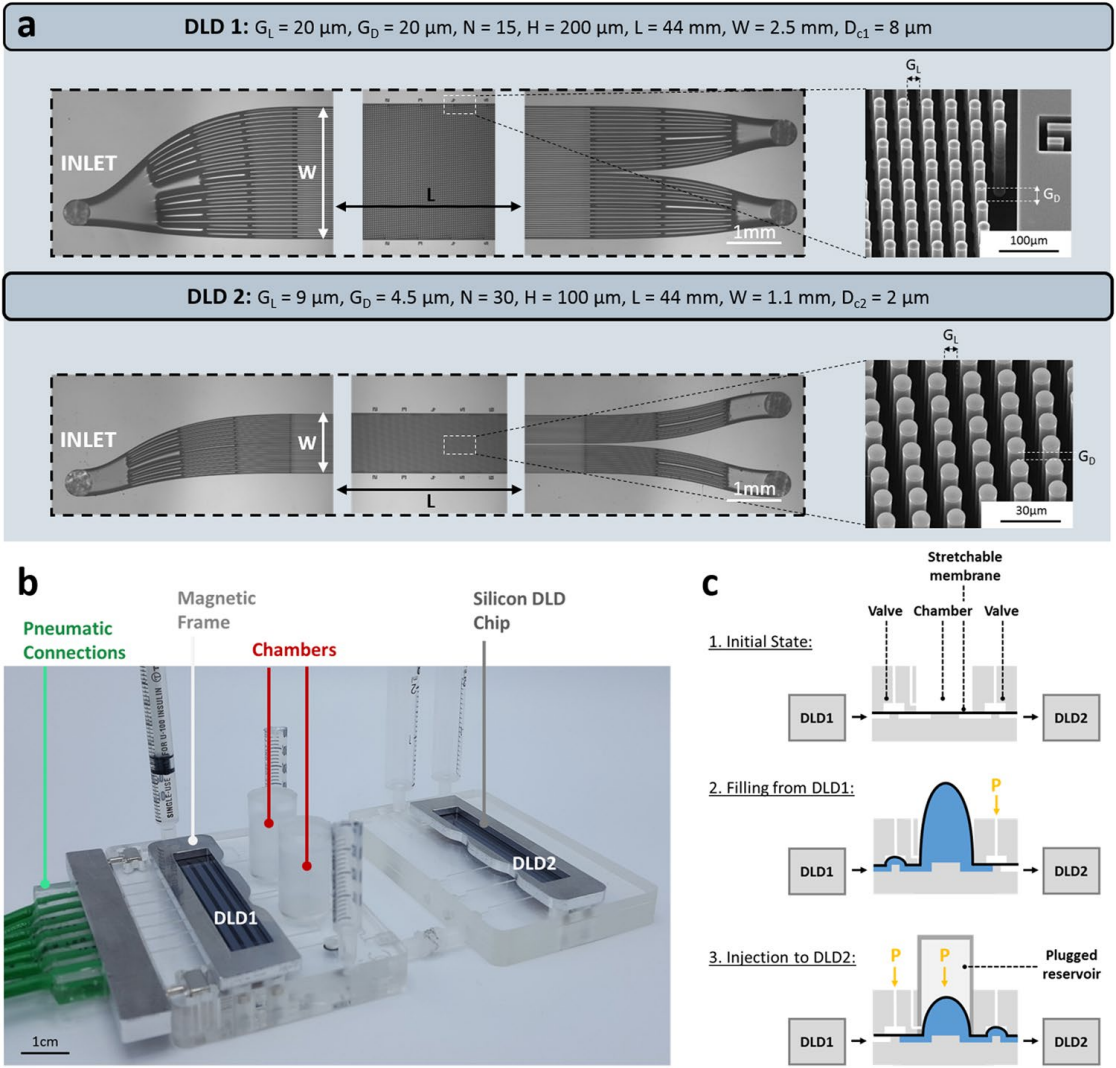
(**a**) Optical and Scanning Electron Microsopy (SEM) images of the DLD1 and DLD2 silicon chips, with corresponding geometric parameters. *G*_*L*_ is the longitudinal gap, *G*_*D*_ is the downstream gap, N is the array period, H is the channel width, L is the channel length, W is the channel width and *D*_*c*_ is the critical diameter. (**b**) Experimental setup with both cascaded DLD modules, called DLD1 and DLD2. DLD1 and DLD2 chips are held on two connected cartridges using magnetic holders. Two deformable chambers covered with solid removable reservoirs are integrated in the DLD1 cartridge to collect samples after the first purification step. A pressure controller enables to precisely actuate both chambers, and a pneumatic connector is magnetically held on the DLD1 cartridge to actuate each valve independently. (**c**) Schematic of the three successive actuation steps of the stretchable membranes. Step 1: the valves are not actuated and the chamber is empty, the stretchable membrane is flat and undeformed. Step 2: The downstream valve is closed by applying pressure on the membrane, while the upstream valve from DLD1 is open to enable the filling of the chamber through the swelling of the stretchable membrane. Step 3: The upstream valve is closed to avoid backflow towards DLD1 and the downstream valve connected to DLD2 is open. Controlled pressure is applied on the chamber using a plugged reservoir in order to inject the sample towards DLD2.

The chips are fabricated using standard microtechnologies on 200 mm silicon wafers. Contact photolithography and Reactive Ion Etching (RIE) are used to define the features of the fluidic channels and ports on a 3 *μ*m-thick silicon dioxide (SiO2) hard mask layer (2 *μ*m thermal SiO2 followed by a deposition of 1 *μ*m SiO2). The fluidic microchannels are then etched through the hard mask using deep UV photolithography and RIE. After removing the oxide by selective wet etching and cleaning the substrate, a second thermal oxide layer (100 nm thick) is grown prior to the sealing of a top 500 *μ*m thick borosilicate glass cover wafer by anodic bonding. Then, the holes are etched from the back side through the silicon substrate to open the fluidic ports until the glass cover. The wafers are then diced into 22 mm × 60 mm individual chips, each containing 6 DLD devices. The devices feature one inlet for sample injection and two outlets to collect non-deviated and deviated particles. Two designs are selected here with the following geometric parameters: lateral gap (GL) = 20 *μ*m, downstream gap (GD) = 20 *μ*m, array periodicity (N) = 15, channel depth (H) = 200 *μ*m, channel length (L) = 44 mm, channel width (W) = 2.5 mm for the first module; and GL = 9 *μ*m, GD = 4.5 *μ*m, N = 30, H = 100 *μ*m, L = 44 mm, W = 1.1 mm for the second module (Fig. 1a). Circular pillars are chosen for both DLD modules. Zhang *et al*.^21^ showed that the pillar shape strongly inflences the behavior of particles in DLD devices, especially for non-isotropic and deformable components. Thus, a comparative study of the deviation of RBCs was performed with circular, triangular, hexagonal and I-shaped pillars (data not shown). More than 90% of injected RBCs were deviated with both circular pillars with asymmetric gaps and I-shaped pillars with symmetric gaps. The circular shape was finally preferred as it provides better bacterial recovery yield (1.6 times higher) compared to I-shaped pillars that probably capture more bacteria in the concave profile.

The position and dimension of the fluidic inlet/outlet holes are standardized so that the same packaging solution can be used for all the different DLD designs^26^. The chips are packaged on a custom COC (Cyclic Olefin Copolymer) cartridge with plug and play tubing connectors (Supplementary Fig. S1). Fluidic sealing between the silicon chip and the plastic cartridge is achieved by a magnetic frame holding silicone-based gaskets. Pneumatic and fluidic channels are micromachined in the cartridge and the different COC sheets of each cartridge are thermally sealed. The valves are made of an EPDM (Ethylene Propylene Diene Monomere, Diacom, EC6508) patch (3.4 mm diameter) that is thermally sealed between two cylindrical apertures. The stretchable membranes for the chambers are prepared by spin coating an Ecoflex 00-50 (Smooth-On, Inc.) rubber to get a final thickness of about 300 *μ*m. This material has been characterized elsewhere^27^. Ecoflex patches are thermally sealed between COC sheets and are free to deform during sample filling through 10.5 mm wide cylindrical openings in the cartridge. Each chamber can contain up to 1 mL of sample. In order to apply controlled pressures on these chambers, removable reservoirs are plugged on top of the cartridge during the injection step.

### Experimental setup

Two cartridges are connected together with plug and play tubing connectors. The first cartridge holds the first DLD chip and the collection chambers with the valves isolating the two DLD stages. The second cartridge holds the second DLD chip. Four different pressure values are controlled independently with a pressure-based flow controller (Fluigent, MFCS-EZ): P_1_ = 100 mbar at the entrance of the first DLD device (resulting in a flow rate of about 200 *μ*L/min), P_2_ = 500 mbar on the chamber collecting the non-deviated sample (resulting in a flow rate of about 30 *μ*L/min in the second DLD device), P_3_ = 200 mbar on the chamber collecting the deviated sample, and P_4_ = 2 bar for valve closing. Valves and chambers are controlled through pneumatic channels integrated in the first cartridge (Supplementary Fig. S2). The closing of each independent valve is automatically controlled by a data acquisition system (USB-1208FS, Measurement Computing). The flow rates in both DLD modules are chosen to obtain a Stokes flow and avoid flow stream mixing, while optimizing the particle separation efficiency (obtained for minimized cell deformation and clogging issues). Acceptable flow rates range from 100 to 600 *μ*L/min for the first DLD module and from 7 to 50 *μ*L/min for the second DLD module.

Imaging is performed by epifluorescence microscopy (Olympus, BX60) with a built-in 100 W mercury lamp (Osram, HBO 103 W/2). A standard FITC (Fluorescein IsoThioCyanate) filter cube (Olympus, U-MSWB2) is used to detect fluorescent beads. Imaging is performed with a digital CMOS camera (Hamamatsu, Flash 4.0 LT+). Sequences of images are superimposed and analyzed with ImageJ software to visualize the trajectory of particles flowing in the DLD channel.

### Sample solutions

Fluorescent polystyrene beads (ThermoFisher Scientific, Fluoro-Max Dyed Green Aqueous Fluorescent Particles) of different sizes (10 *μ*m, 5 *μ*m and 1 *μ*m) are used to characterize the DLD devices, by modeling the three characteristic sizes of our biological samples (PC3 cells that display sizes from 13 to 19 *μ*m, RBCs with diameters from 6 to 8 *μ*m and thicknesses from 1 to 2.5 *μ*m and *E. coli* bacteria with a width of 0.8 *μ*m and a length distribution between 2 and 5 *μ*m). As recommended by the manufacturer, the beads are suspended at 106/mL in a 1% surfactant solution (Tween 20, Sigma-Aldrich) in filtered DPBS 1x (gibco life technologies, 14190144) in order to prevent particle aggregation.

PC3 cells (ATCC, CRL-1435) are transfected with pEGFP-C1 plasmids. PC3-GFP cells (~80% confluent) are harvest in trypsin (0.05% Trypsin-EDTA, Gibco, 25300-054) and resuspended in fresh medium (RPMI 1640-Gibco, 10% Fetal Bovine Serum, 1% Penicillin-Streptomycin, 0.5% Geneticin).

Blood samples are collected from healthy donors (Etablissement FranÃ§ais du Sang (EFS), Grenoble, FRANCE) in EDTA BD Vacutainer tubes and diluted in a ratio of 1:10 in DPBS 1x. Informed consent was given by blood donors, according to the ethical and legal standards of EFS. Blood usage was regulated by the Health Department of Research Ministry, according to the French directive DC-2008-334. Blood tubes were delivered 3 days after withdrawal and were used for experiments at room temperature the same day as delivery. After 10x dilution in DPBS 1x, we verify that the hematocrit is between 4 and 5% for all blood experiments.

The Escherichia coli GFP (ATCC, 25922GFP) strain is grown in ampicillin-treated Luria Bertani (LB) agar plates at 37 °C in 5% CO_2_ and incubated overnight in LB agar plates without ampicillin to avoid filamentous forms of *E. coli* bacteria that would induce variabilities in bacterial dimensions. Therefore, in our experiments, bacteria display the same dimensions as wild *E. coli* strains grown in LB agar plates^28^.

PC3 cells are resuspended at 5 × 10^6^ cells/mL in 2 mL of 10x-diluted blood, after centrifugation at 300 × g for 5 minutes. Bacteria are scraped from the plates and resuspended in the 10 × -diluted blood sample containing PC3-GFP cells to a final concentration of 1.0 × 10^6^–1.0 × 10^7^ bacteria/mL.

The concentration of particles in initial samples and output solutions after experiments is quantified by optical counting of at least 400 particles per condition with Kitvia Cell Fast-Read plates (fisherscientific, H01BVS100). For each condition, a sample of at least 500 *μ*L is collected without any problematic clogging.

## Results and Discussion

### Two-stage separation principle

We present a two-stage purification platform with two successive DLD modules, called respectively “DLD1” and “DLD2” (Fig. 1b). The sample is first injected in DLD1 to separate particles around the critical diameter *D*_*c*1_ = 8 *μ*m. Each DLD1 outlet is connected to a chamber. The inlet of DLD2 is connected to the chamber that contains non-deviated particles from DLD1. DLD2 separates particles around a critical diameter *D*_*c*2_ = 2 *μ*m, that was determined from our experiments with different bead sizes.

The chambers are made of a hyperelastic membrane that is initially pressed against one of the cartridge sheets and then progressively swells to get filled with the sample flowing out from DLD1 (Fig. 1c). When the membrane reaches its maximal deformation amount - which corresponds to a chamber filling of about 1 mL - air pressure is applied on top of the membrane to press it down and inject the sample towards a downstream fluidic channel. Similar membranes were implemented by Parent *et al*.^29^ to control liquid volumes of up to 100 *μ*L. Here we have adapted this technology to achieve a storage volume of up to 1 mL in our flexible chambers, using openings in the cartridge that allow free deformation of the membrane (Fig. 1c). Therefore, in our case, the maximum filling volume is only limited by the deformation before rupture of the membrane. In order to apply high pressures on the membrane, a removable reservoir is plugged on the chamber opening, resulting in a closed controlled environment around the chamber (shown in Fig. 1b and schematically represented for the third step in Fig. 1c). The advantage of these stretchable membranes compared to rigid chambers is that they address the issues of air bubble injection during filling in the second DLD stage. Indeed, no air is introduced in the chamber when pressure is applied on the membrane, since the pneumatic channel controlling injection is located on the opposite side of the membrane compared to the fluidic channels (Fig. 1c).

The two-stage purification protocol is composed of successive automatically controlled steps. First, the sample is injected in DLD1 and the chambers are filled with the non-deviated and deviated samples. Both chambers fill up simultaneously with the same amount of sample since our DLD modules have been designed to allow the sample to flow out from both outlets at the same flow rate. The swelling of both membranes during sample injection in the first DLD chip is illustrated by Supplementary Video S1. Valves are placed upstream and downstream of both chambers to achieve purification steps successively. First, the downstream valves (between the chambers and DLD2) are closed, while the upstream valves (between DLD1 and the chambers) are open to store the samples from DLD1 in the chambers and perform DLD1 purification without any disturbance from DLD2 (Step 2 in Fig. 1c). When both chambers are filled with about 1 mL of sample, injection at the DLD1 entrance is stopped and the upstream valves between DLD1 and the chambers are closed. The deviated sample can be collected for further analysis simply by opening the valve placed downstream of the deviated chamber (Supplementary Video S2). The non-deviated sample is then injected in DLD2 by closing the upstream valve connected to DLD1 and opening the downstream valve connected to DLD2 (Step 3 in Fig. 1c). An alternative downstream valve enables to collect or remove the non-deviated sample without passing through DLD2 (Supplementary Fig. S2). Sample injection in DLD2 requires to apply pressure on the membrane owing to the high hydraulic resistance presented downstream. This is why a solid reservoir is plugged on the cartridge above the membrane and connects it to a pneumatic channel from the cartridge. We verify the filling of both DLD2 outlets (to output syringes) while applying pressure on the non-deviated chamber in the plugged reservoir (Supplementary Video S3). At the end of the protocol, particles smaller than *D*_*c*2_ are extracted from particles larger than both *D*_*c*1_ and *D*_*c*2_.

The DLD1 module integrates the chip, both output chambers, the valves and their pneumatic actuation channels and connectors (Supplementary Fig. S1). Fluidic sealing of the chip on the cartridge is performed by a plug-and-play magnetic holder and silicone gaskets. The cartridge consists of four different levels: a first level (1 on Supplementary Fig. S1) that contains both chamber openings and pneumatic channels to actuate the valves and chambers located in the second level (2 on Supplementary Fig. S1). The valve and chamber membranes are sealed between levels 2 and 3 of Supplementary Fig. S1 and are connected to the fluidic channels of the bottom layer (represented in the enlarged box of Supplementary Fig. S1). These fluidic channels connect the DLD1 outlets to the chambers and to the valves placed upstream and downstream of each chamber. Pressure is controlled through the pneumatic channels with the help of a lateral connector, that is maintained against the layer n^o^1 with magnets.

Thus, integrated valves and collection chambers between both DLD modules enable automatic control of successive DLD purification steps without any intermediary sample manipulation. Sequential operation of the DLD steps is demonstrated for the first time using our integrated flexible chambers, while previous work used either a single chip or external flow rate balancing with syringe pumps or droplets.

### Validation of the two-stage fractionation with beads

**Figure 2:**
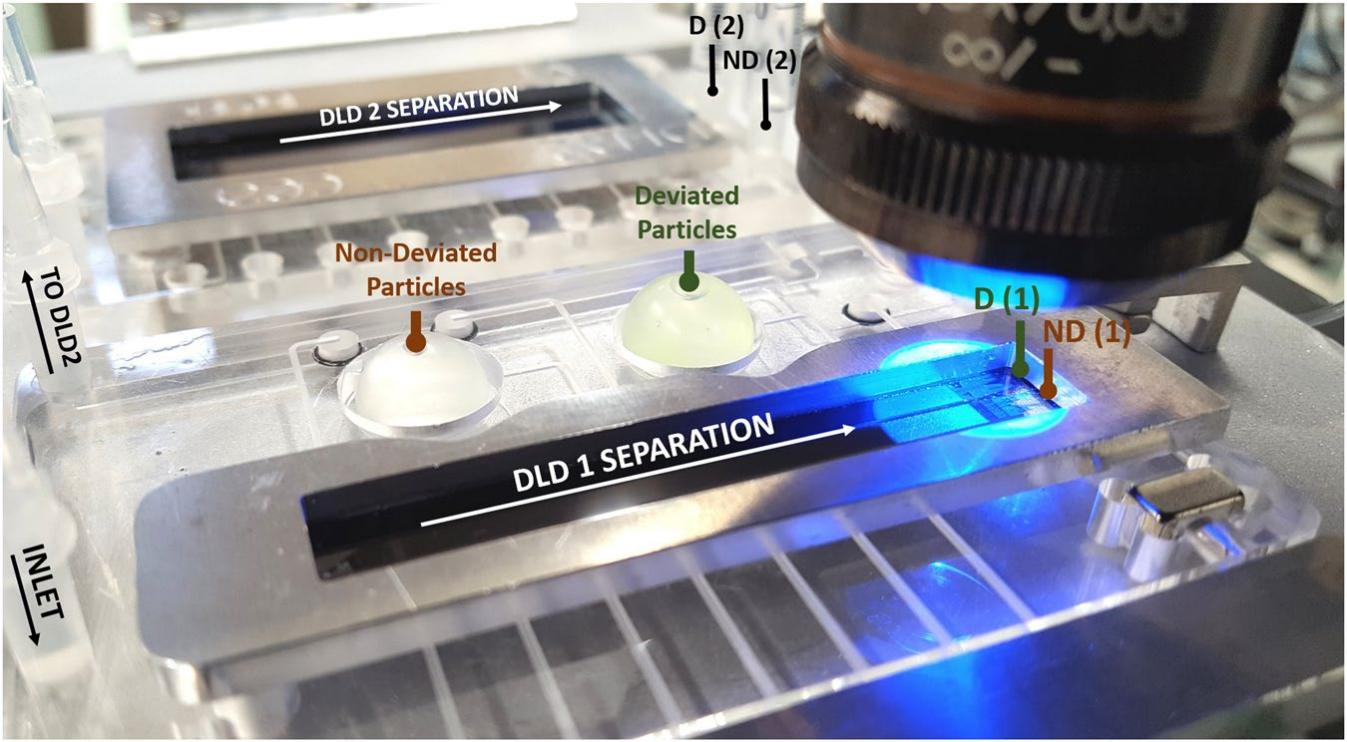
Cascaded DLD devices during testing with model microbeads. A mixing of 10 *μ*m, 5 *μ*m and 1 *μ*m-beads is injected through the inlet of the DLD1 module and the flexible chambers are filled respectively with the non-deviated particles (flowing out through the DLD outlet called ND) and with the deviated particles (flowing out through the DLD outlet called D). The non-deviated particles are then injected in the DLD2 module for further separation.

**Figure 3:**
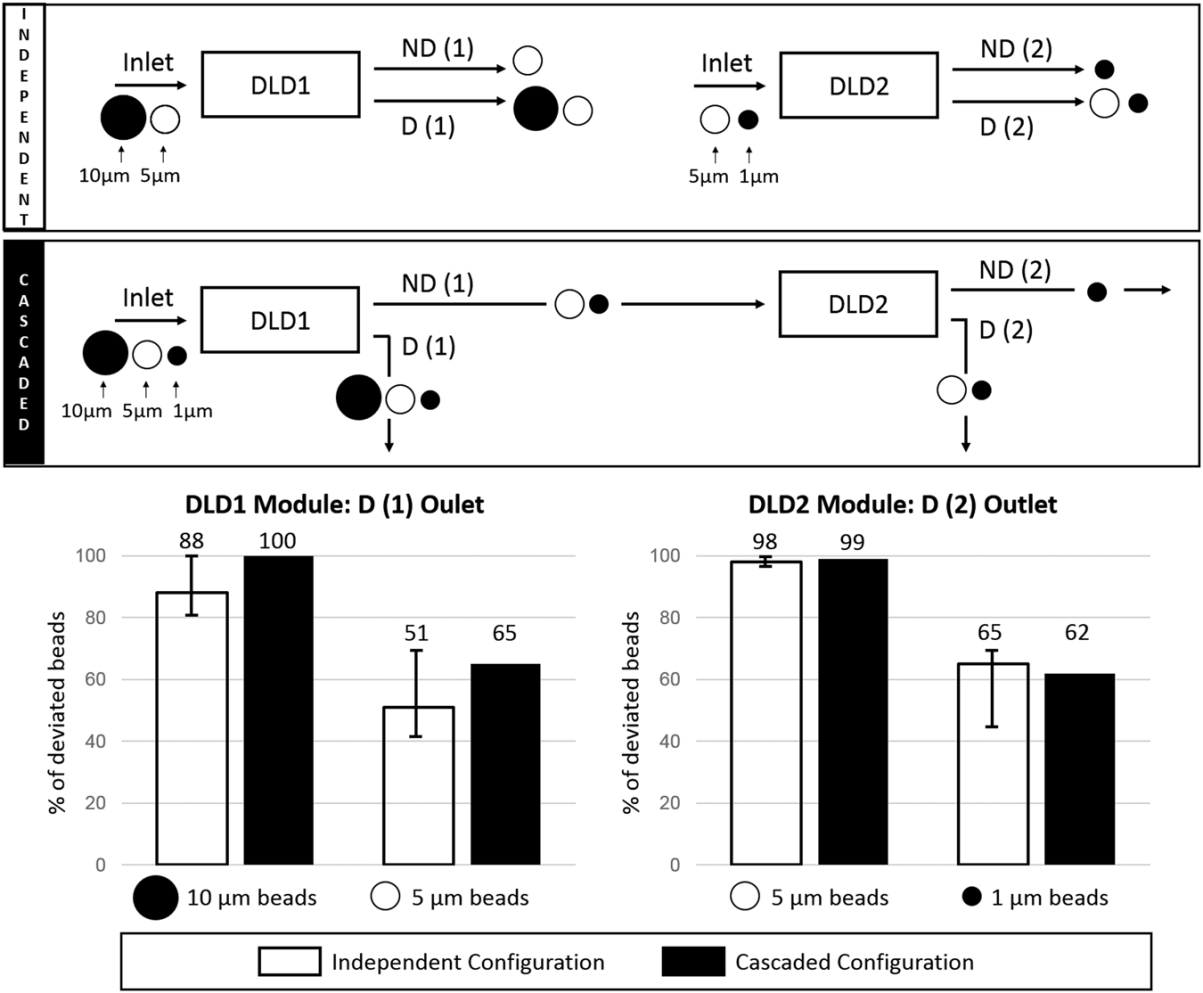
Schematic representation of the compared independent and cascaded configurations to separate 10 *μ*m, 5 *μ*m and 1 *μ*m-beads. The deviated outlets (D (1) for DLD1 and D (2) for DLD2) are analyzed in both cases to quantify the ratio of deviated beads among the total output particles for each bead size.

First, we validated the developed integrated platform for cascaded separation with model microbeads. A mixture of 10 *μ*m, 5 *μ*m and 1 *μ*m-beads is used as a model sample for two successive separations around the respective critical diameters *D*_*c*1_ = 8 *μ*m and *D*_*c*2_ = 2 *μ*m. The sample is injected through the DLD1 inlet (Fig. 2) and flows through the DLD1 chip. Non-deviated (ND) beads are collected in the left chamber of Fig. 2, while deviated (D) beads are simultaneously collected in the other chamber. We observe that the deviated chamber displays a light green shade compared to the non-deviated chamber (Fig. 2), which is attributed to the presence of larger beads, with higher fluorescence intensity. Then, non-deviated beads are injected in the DLD2 module and this second purification step enables to collect two bead populations in the DLD2 outlets called ND (2) and D (2) on Fig. 2. In order to simplify the fluidic control at the channel entrance, our DLD devices feature only one inlet. Thus, the injected particles span the full width of the channel at the entrance of the device. Therefore, in the case of particles smaller than the cutoff diameter *D*_*c*_, at least 50% of the injected beads should actually flow out through the deviated outlet, since they display a non-deviated trajectory (Fig. 3). Conversely, all particles larger than *D*_*c*_ should be deviated. We experimentally validated this capacity, and observed that the entire population of large particles - including those initially injected in the non-deviated side - are retrieved at the deviated outlet.

**Figure 4:**
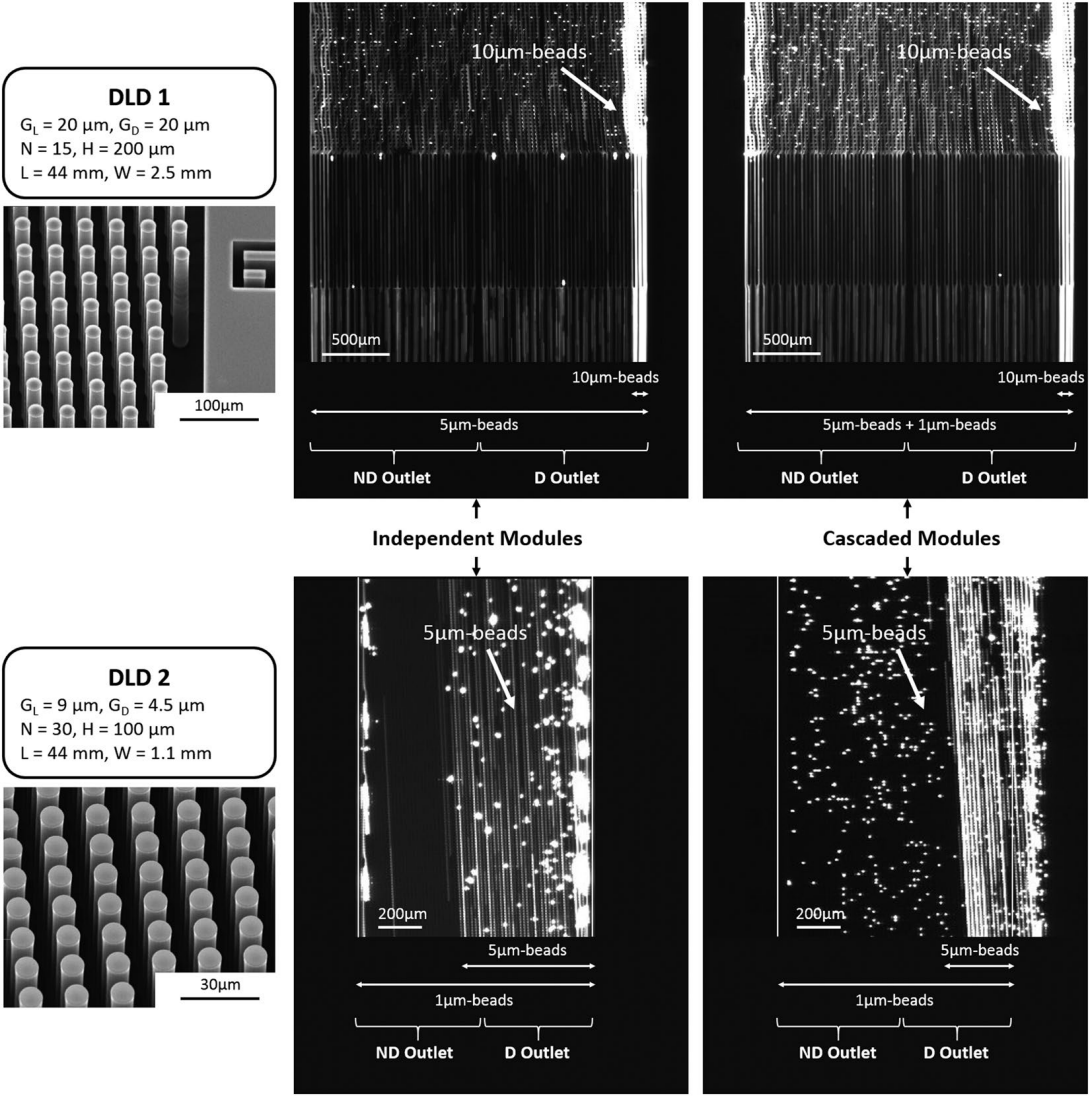
Fluorescent images of the trajectory of microbeads in the DLD1 and DLD2 modules in both the independent (left images) and cascaded (right images) configurations. The outlet position of the non-deviated (ND) and deviated (D) outlets is given for each observed channel. The geometrical parameters and SEM images are also given for each module. *G*_*L*_ is the longitudinal gap, *G*_*D*_ is the downstream gap, N is the array period, H is the channel width, L is the channel length and W is the channel width. White deviated streaks correspond to 10 *μ*m-beads in DLD1 and 5 *μ*m-beads in DLD2.

During DLD sorting, we monitor the trajectory of the fluorescent particles in both modules (Fig. 4). The results obtained with the cascaded platform are compared to the separation efficiency of each independent module. Images at the outlet of DLD1 clearly show intense fluorescent streaks on the right side, that correspond to deviated 10 *μ*m-beads, while 5 *μ*m-beads flow out through the entire channel width since they are not deviated from the single inlet. No significant difference can be noticed in the deviation efficiency between the independent and the cascaded configurations, which is confirmed after quantification of the beads in both DLD1 outlets (Fig. 3). Indeed, 88% of the collected 10 *μ*m-beads are detected in the deviated outlet in the independent configuration, while the cascaded configuration leads to the deviation of 100% of the collected 10 *μ*m-beads. In the channel of DLD2 (Fig. 4), white deviated streaks corresponding to the trajectory of 5 *μ*m-beads are observed. Intense fluorescent streaks near the channel walls can be seen when beads are captured on both sides, which is particularly important in DLD2 since the downstream gap is smaller than the 5 *μ*m bead diameter. We verify that more than 98% of the collected 5 *μ*m-beads are deviated by Module 2 in both the independent and the cascaded configurations (the zigzag path is not possible in this case for 5 *μ*m-beads given the 4.5 *μ*m downstream gap), while 1 *μ*m-beads are also partly deviated by Module 2, with 62 to 65% of them counted in the deviated outlet (Fig. 3). It should be pointed out that the percentages given for DLD2 in the cascaded configuration are relative to the number of 5 *μ*m and 1 *μ*m-beads that are collected in the non-deviated outlet of DLD1 (about 50% of the beads initially injected in DLD1 because of the one-inlet/two-outlets configuration).

We also verify that the recovery yield (defined as the percentage of injected particles that are collected at the DLD outlets) is not degraded in the cascaded configuration, which confirms that the beads are efficiently injected out of the flexible chambers. The obtained recovery yield at the DLD1 stage was 100% in both the independent and the cascaded configurations for the 10 *μ*m and 5 *μ*m beads. The DLD2 module displays recovery yields of 68% and 80% for 5 *μ*m beads, and 65% and 70% for 1 *μ*m beads, respectively in the independent and the cascaded configurations. Thus, our cascaded platform displays similar performances as independent modules to successively separate particles around two different critical diameters.

### Bacteria isolation from blood samples with the autonomous platform

**Figure 5:**
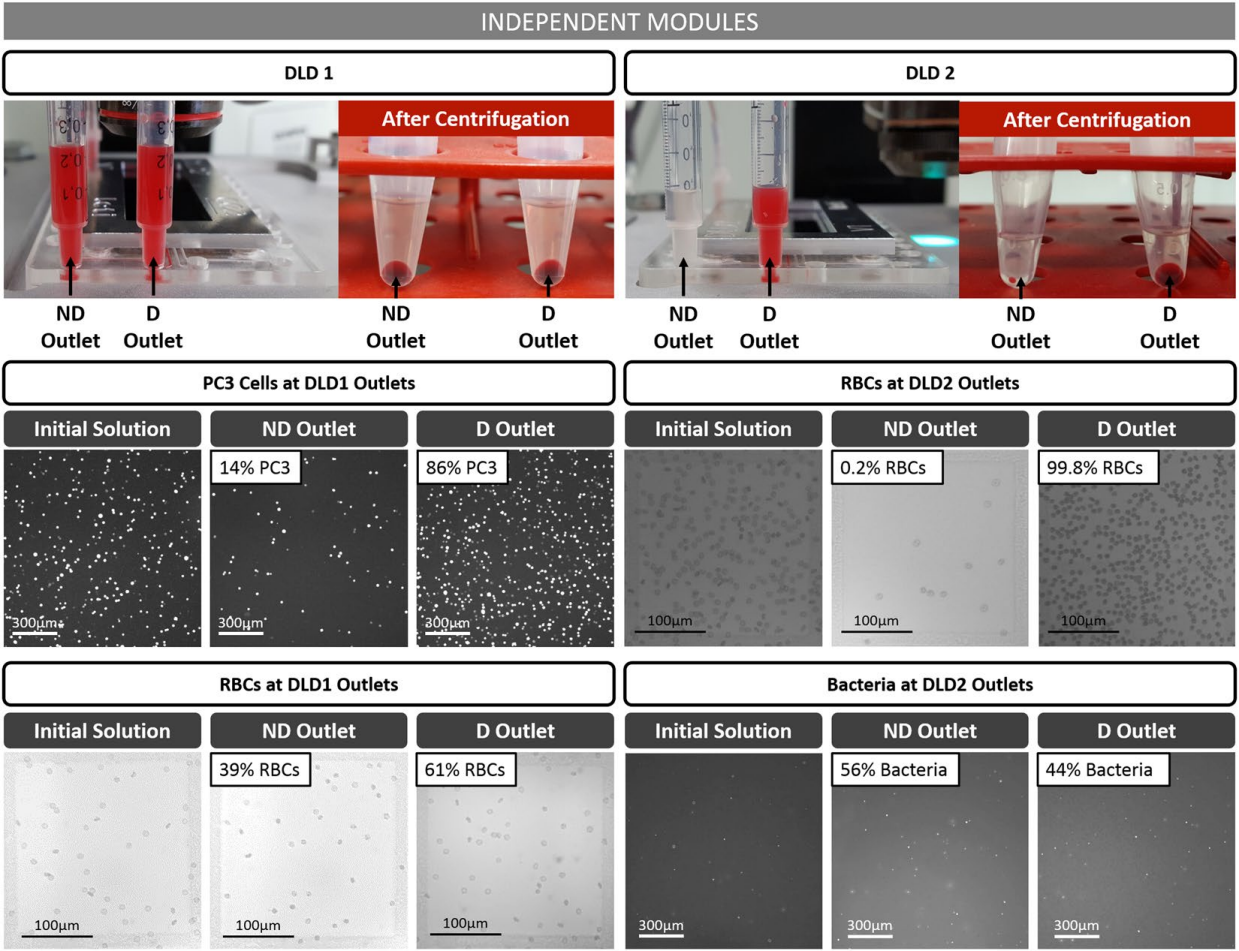
Independent modules: DLD outlets of DLD1 and DLD2 before and after centrifugation. Quantification of PC3-GFP cells (epifluorescence image), RBCs (bright-field image) and *E. coli* GFP bacteria (epifluorescence image) in the initial sample solution and in both outlets of DLD1 and DLD2. DLD1 deviates 86% of the collected PC3 cells and 61% of the collected RBCs, while DLD2 enables the deviation of more than 99% of the collected RBCs.

We implemented the same cascaded platform to fractionate a complex biological sample, containing three main particle dimensions: PC3 human prostate cancer cells, blood cells and *E. coli* bacteria. A two-step purification is required in this case to isolate bacteria and remove both PC3 cells and blood contaminants (mainly RBCs). PC3 cells were GFP-transfected to enable to follow their trajectory in DLD modules. The number of particles in each sample was determined on counting plates, in fluorescence microscopy for PC3 cells and GFP-modified bacteria, and in bright-field microscopy for RBCs. We first validated both DLD1 and DLD2 modules independently (Fig. 5): DLD1 deviates 61% of the collected RBCs and removes 86% of PC3 cells, while DLD2 removes 99.8% of RBCs. As expected from the critical diameter of DLD2 (*D*_*c*2_ = 2 *μ*m), bacteria are not deviated (suggesting that they are oriented along their smallest dimension while flowing along DLD pillars) and can be found at similar concentrations in both DLD outlets since the sample is initially injected through a single inlet. Since white blood cells (WBCs) were not labeled, their trajectory and distribution could not be visualized, but most of them are expected to be deviated by DLD1 (*D*_*c*1_ = 8 *μ*m), since their dimensions range from 7 to 20 *μ*m^30^. Moreover, given the low concentration of WBCs relative to PC3 cells in our 10x-diluted blood samples^31^, the obtained results should not be influenced by the behavior of WBCs.

**Figure 6:**
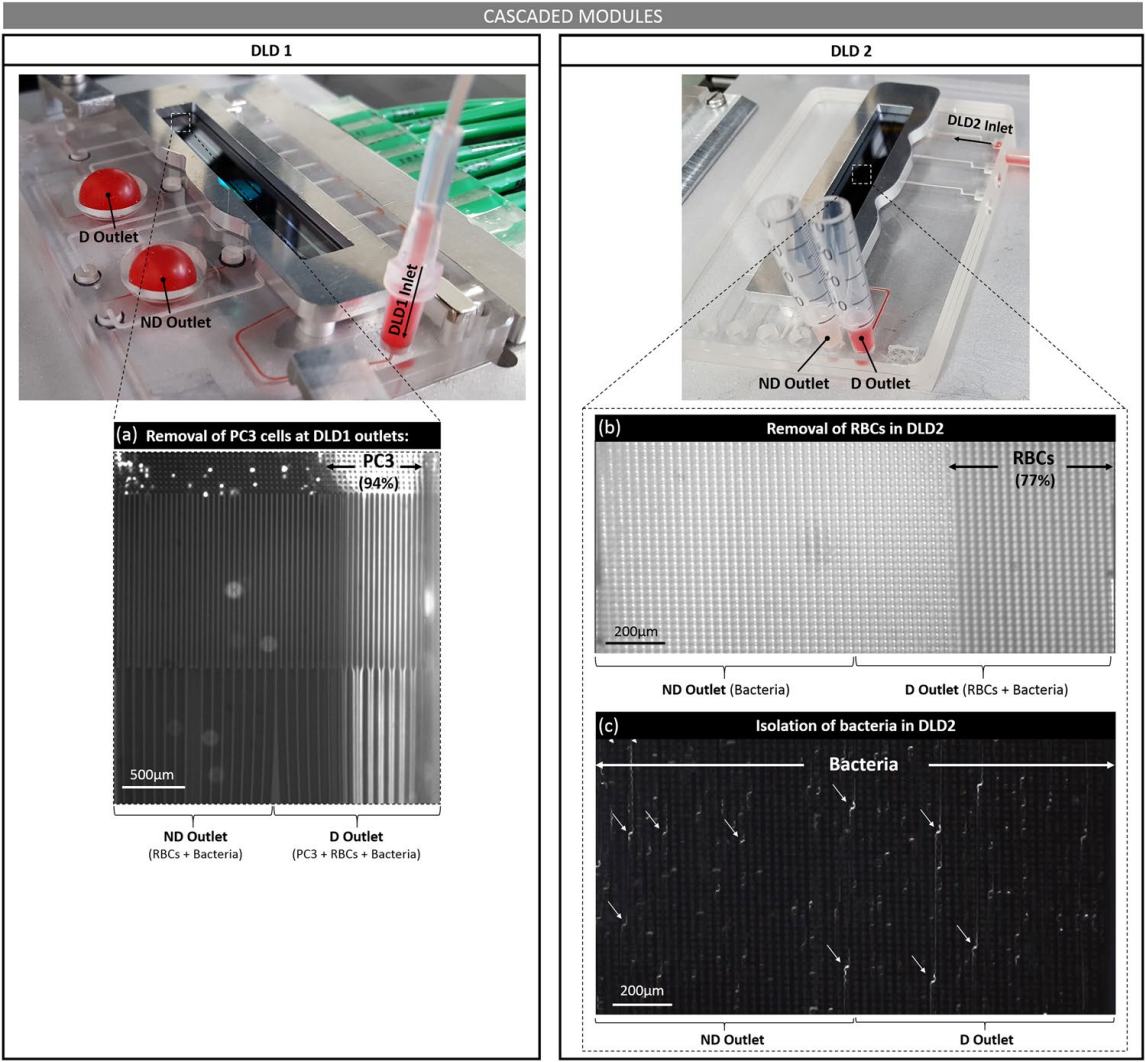
Cascaded modules: Images of both modules with the corresponding trajectory of deviated PC3 cells at DLD1 outlets (**a**), deviated RBCs in DLD2 (**b**) and non-deviated bacteria in DLD2 (**c**). Periodic zigzag displacements of bacteria are pointed by white arrows.

**Figure 7:**
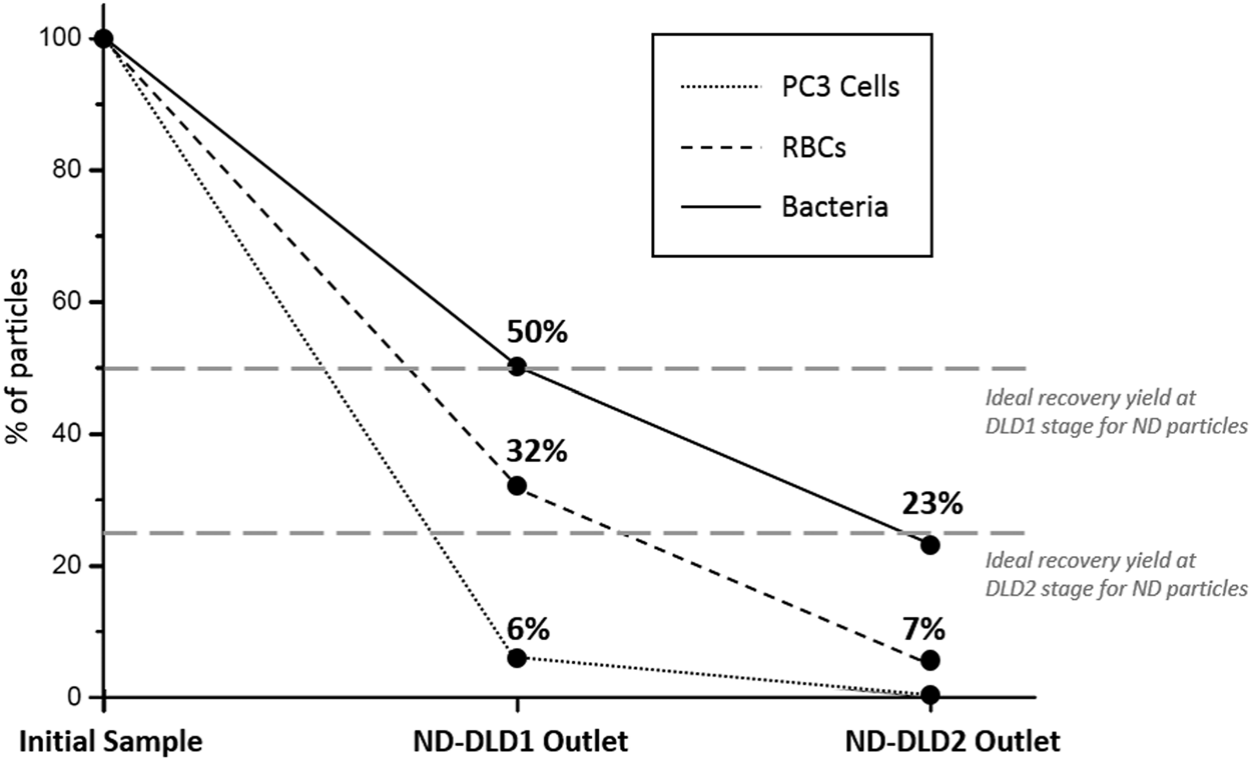
Cascaded modules: Quantification of residual non-deviated PC3 cells and RBCs, as well as collected non-deviated *E. coli* bacteria, at each DLD purification stage. 94% of PC3 cells and 68% of RBCs are removed by DLD1, while 77% of RBCs are eliminated by DLD2. In the final non-deviated channel of DLD2, that contains our sample of interest, 23% of bacteria are depleted from 100% of the initial PC3 cells and 93% of RBCs.

Both modules were then connected together in the cascaded platform. Figure 6 shows the chambers at the outlet of Module 1 respectively filled with the non-deviated (ND) and deviated (D) particles. PC3 cells are clearly focused on the deviated side of DLD1 from fluorescent imaging of the outlet (Fig. 6a). Similarly to the independent configuration, Module 1 enables depletion of 94% of the collected PC3 cells, while 68% of RBCs are removed by the first DLD stage (Fig. 7). Images of DLD2 show the focus of RBCs on the right deviated side of the channel and the zigzag trajectory of GFP-modified *E. coli* bacteria (Fig. 6b,c). We verify that 77% of RBCs flowing out of DLD2 are collected in the deviated outlet, while 46% of output bacteria are not deviated by DLD2 (Fig. 7).

Therefore, after both purification steps through the cascaded platform, the non-deviated outlet of Module 2 contains more than 20% of the initially injected *E. coli* bacteria with less than 8% of the initial number of RBCs and 0% of PC3 cells. The recovery yield of bacteria after both DLD purification steps closely approaches the ideal 25% recovery yield (Fig. 7), owing to our one-inlet/two-outlets configuration at each step. This final recovery yield could still be improved by using several cascaded channel depths, as proposed by Holm *et al*.^32^, or with DLD devices with two inlets instead of the single entrance configuration used in this paper. DLD devices with two inlets would enable to reach 100% recovery of the non-deviated particles although it requires balancing control of both injected solutions (sample and buffer) at the channel inlets.

## Conclusion

We presented a two-stage approach to automatically cascade several DLD purification steps in a single integrated platform. As a result of the modularity of our cascaded platform, any DLD chips can be implemented according to the application. Intermediate flexible chambers with high volume capacitance are directly integrated in the cartridge and present two advantages: each DLD step is performed independently with optimized separation efficiency - similar to single DLD devices - and no external manipulation of the sample is required. Each step of the purification protocol is performed automatically, with the help of external actuation of valves and chambers controlling the sample flow between both DLD modules. Therefore, the complexity of the fluidic actuation at each DLD stage is integrated in our autonomous cartridge, unlike usual approaches for which it is manually controlled by the user.

This new approach was demonstrated with the extraction of bacteria from complex blood samples to achieve a concentration of bacteria three times higher than the most abundant contaminant (RBCs). A two-stage device was implemented for this application but more than two DLD steps could be cascaded with the exact same strategy. The presented platform with integrated large-volume chambers opens a perspective of automatically controlling any successive microfluidic steps that need to be performed independently from each other to ensure optimized efficiency.

## Electronic supplementary material

Supplementary Information

Supplementary Video S1

Supplementary Video S2

Supplementary Video S3

## Data Availability

The authors declare that materials, data and associated protocols are promptly available to readers without undue qualifications in material transfer agreements.
