# Supplementary material for: Separation of Biological Particles in a Modular Platform of Cascaded Deterministic Lateral Displacement Modules
Source: Sci Rep. 2018 Dec 10;8:17762. doi: 10.1038/s41598-018-34958-8 (PMC6288093; doi:10.1038/s41598-018-34958-8)
Supplement: Supplementary file 1 — Supplementary Information [file 41598_2018_34958_MOESM1_ESM.pdf]

## Title

Separation of Biological Particles in a Modular Platform of Cascaded Deterministic Lateral Displacement Modules


## Authors / Affiliations

Eloise Pariset[1], Charlotte Parent[1], Yves Fouillet[1], François Boizot[1], Nicolas Verplanck[1], Frédéric Revol-Cavalier[1], Aurélie Thuaire[1], and Vincent Agache[1]

[1] Univ. Grenoble Alpes, CEA, LETI, DTBS, F-38000 Grenoble

## Corresponding Author

Vincent Agache, vincent.agache@cea.fr

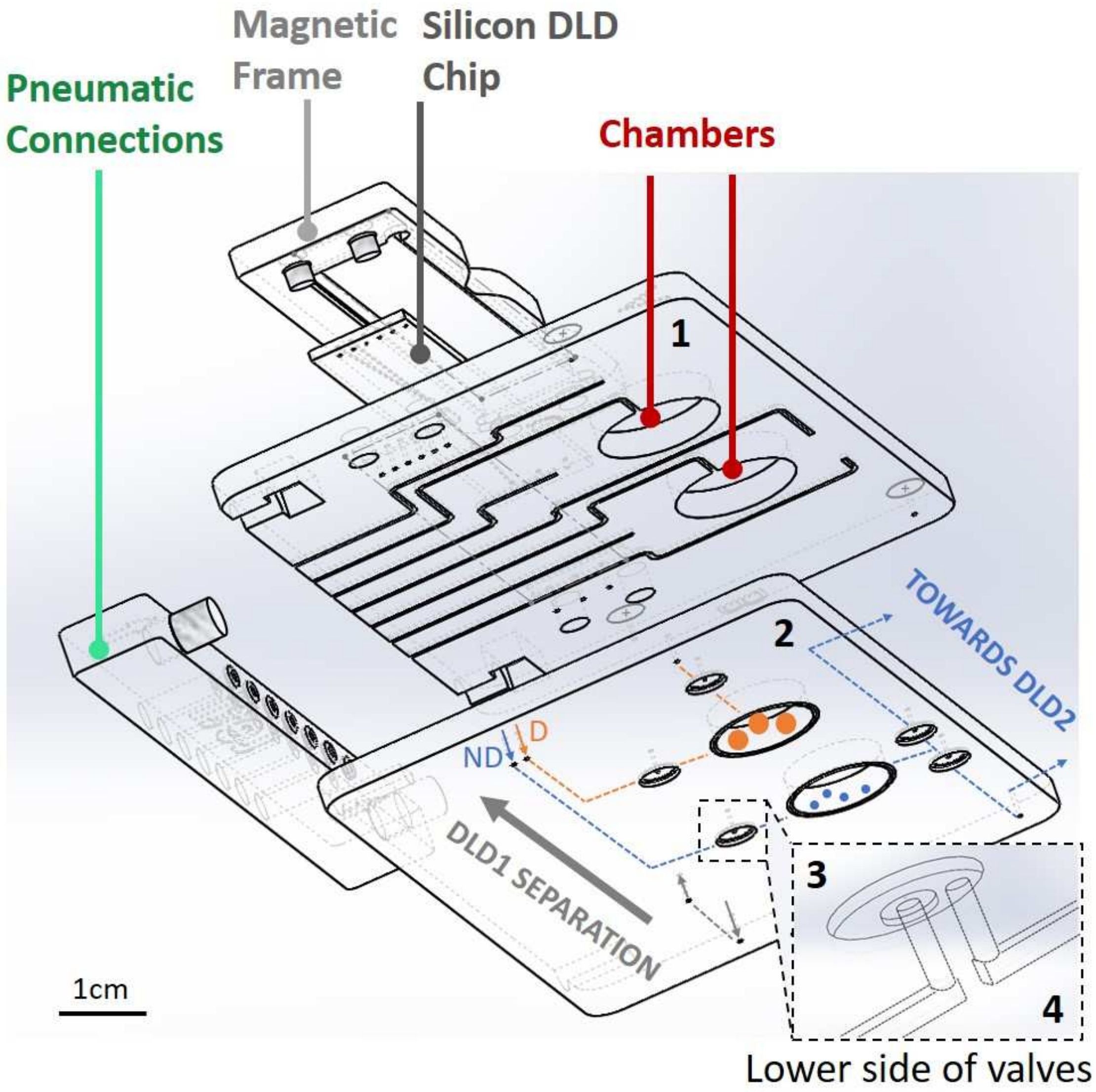


**Supplementary Figure S1:** Exploded view of the first module (DLD1) with pneumatic connections to actuate the valves, magnetic holding of the silicon DLD chip and the four layers of the plastic cartridge: pneumatic channels and chamber openings (layer 1), upper side of the valves and chamber openings (layer 2), lower side of the valves (layer 3 in the enlarged box), fluidic channels (layer 4 in the enlarged box).

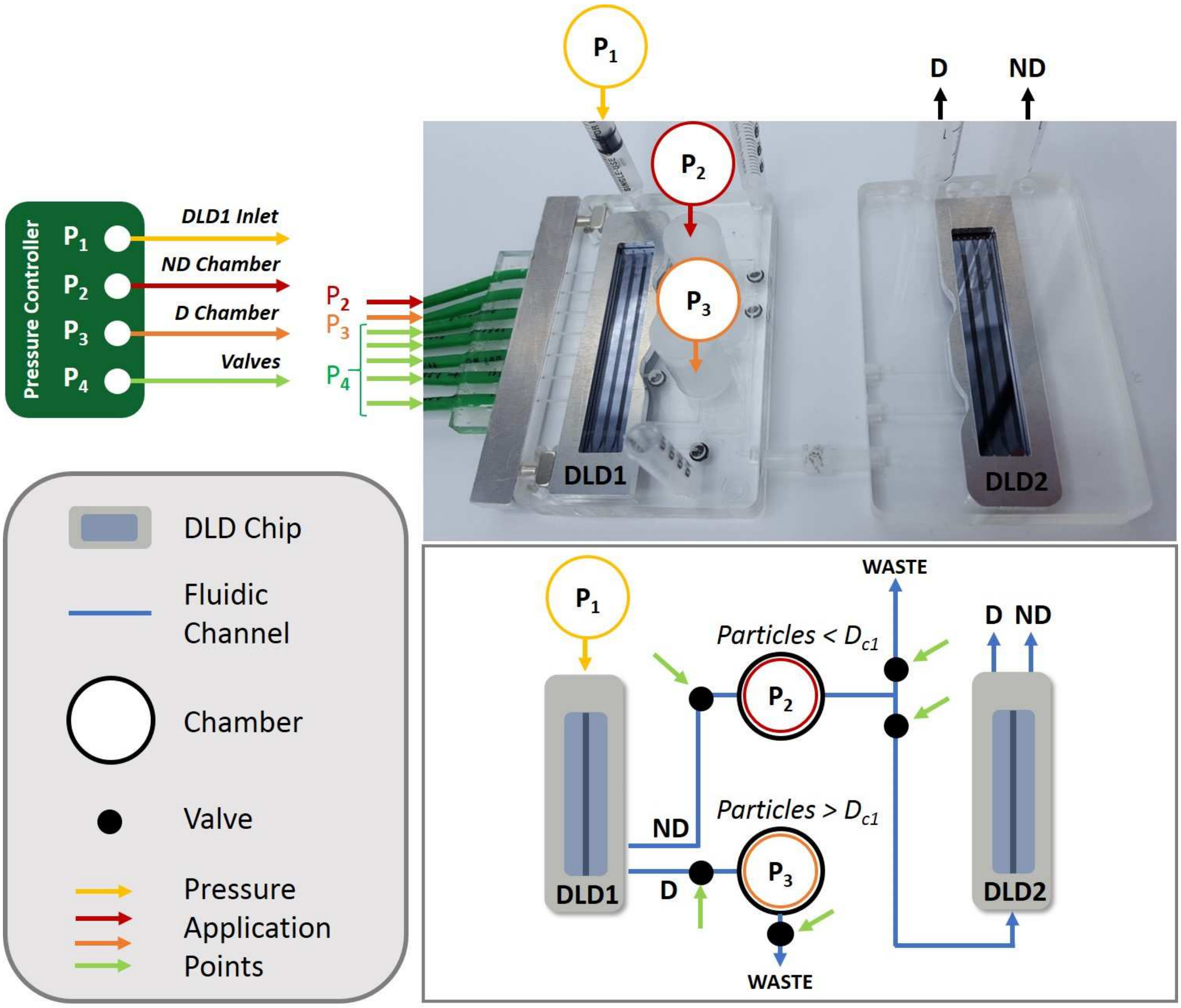


**Supplementary Figure S2:** Picture of the cascaded platform and corresponding schematic representation of the fluidic and pneumatic elements. The pressure application points are given for the DLD1 inlet ($P_1$ = 100 mbar), non-deviated DLD1 output chamber ($P_2$ = 500 mbar), deviated DLD1 output chamber ($P_3$ = 200 mbar) and valves ($P_4$ = 2 bar).
